# CrJAT1 Regulates Endogenous JA Signaling for Modulating Monoterpenoid Indole Alkaloid Biosynthesis in *Catharanthus roseus*

**DOI:** 10.3390/genes15030324

**Published:** 2024-03-02

**Authors:** Mengxia Zhang, Bingrun Yang, Yanyan Wang, Fang Yu

**Affiliations:** 1 School of Biological Engineering, Dalian Polytechnic University, Dalian 116034, China; mxzhang_dlpu@163.com (M.Z.); yangbr185855@163.com (B.Y.); wang_yy@dlpu.edu.cn (Y.W.); 2 College of Bioscience and Biotechnology, Shenyang Agricultural University, Shenyang 110866, China

**Keywords:** *Catharanthus roseus*, gene expression, jasmonic acid signaling, jasmonate transport, monoterpenoid indole alkaloids biosynthesis

## Abstract

Many monoterpenoid indole alkaloids (MIAs) produced in *Catharanthus roseus* have demonstrated biological activities and clinical potential. However, their complex biosynthesis pathway in plants leads to low accumulation, limiting therapeutic applications. Efforts to elucidate the MIA biosynthetic regulatory mechanism have focused on improving accumulation levels. Previous studies revealed that jasmonic acid (JA), an important plant hormone, effectively promotes MIA accumulation by inducing the expression of MIA biosynthesis and transport genes. Nevertheless, excessive JA signaling can strongly inhibit plant growth, decreasing MIA productivity in *C. roseus*. Therefore, identifying key components balancing growth and MIA production in the JA signaling pathway is imperative for effective pharmaceutical production. Here, we identify a homolog of the jasmonate transporter 1, CrJAT1, through co-expression and phylogenetic analyses. Further investigation demonstrated that CrJAT1 can activate JA signaling to promote MIA accumulation without compromising growth. The potential role of CrJAT1 in redistributing intra/inter-cellular JA and JA-Ile may calibrate signaling to avoid inhibition, representing a promising molecular breeding target in *C. roseus* to optimize the balance between growth and specialized metabolism for improved MIA production.

## 1. Introduction

Jasmonic acid (JA) is an oxylipin plant hormone that coordinates various physiological processes crucial for plant survival and stress adaptation [1]. As a critical signaling molecule, JA regulates numerous developmental processes, including growth, differentiation, and response to biotic and abiotic stresses [2]. Upon perceiving threats such as herbivory, pathogen infection, or changes in environmental conditions, biosynthesis of JA is rapidly induced to initiate plant defense mechanisms against stresses [3,4]. In response to JA signaling, many specialized metabolites important for plant defense and stress tolerance are synthesized by activating their biosynthetic pathways [5]. Via its regulation of specialized metabolism, JA activation is critical for protecting plants and enhancing their resilience to diverse stresses [6]. Thus, JA-mediated metabolism adjustments are crucial for plant survival when faced with biotic and abiotic environmental challenges.

In plants, the biosynthesis of JA is initiated in chloroplasts, where linolenic acid is released from membrane lipids. The oxygenation of linolenic acid by lipoxygenase (LOX) leads to the formation of 13(S)-hydroperoxylinolenic acid (13-HPOT) [7]. 13-HPOT is then metabolized sequentially by allene oxide synthase (AOS) and allene oxide cyclase (AOC) to generate cyclopentenone 12-oxo-phytodienoic acid (OPDA) [8,9]. OPDA is transported to peroxisomes, where it is reduced by OPDA reductase (OPR) to form JA [10]. Finally, the bioactive jasmonoyl-isoleucine (JA-Ile) hormone is synthesized from JA by jasmonoyl isoleucine synthase (JAR1) in the cytoplasm [11]. Biotic and abiotic stresses could activate the JA biosynthesis pathway, leading to the accumulation of JA/JA-Ile and initiation of JA signaling. JA-Ile is transported into the nucleus to promote the F-box protein coronatine insensitive1 (COI1) binding to the JAZ transcriptional repressors. This binding leads to ubiquitin-mediated degradation of JAZ proteins and de-repression of the JA signaling pathway [12,13]. Thus, JA biosynthesis and JA-Ile-mediated relief of JAZ repression enable the activation of JA-responsive genes during plant stress responses.

Efficient activation of the JA signaling pathway requires both the biosynthesis of JAs and translocation of the bioactive JA-Ile from the cytoplasm to the nucleus. Recently, an ABC transporter named AtJAT1/AtABCG16 was found to control the subcellular distribution of both JA and JA-Ile in *Arabidopsis thaliana* [14]. AtJAT1 mediates the nuclear import of JA-Ile across the nuclear envelope and the efflux of JA across the plasma membrane. This dual transport function can influence the equilibrium between JA-Ile synthesis versus turnover, enabling dynamic regulation of jasmonate responses in planta. The presence of AtJAT1 enables plants to autonomously activate or deactivate JA signaling, balancing growth and defense processes.

Numerous specialized metabolites have been functionally identified to combat biotic or abiotic stresses in response to JA signaling in plants. *Catharanthus roseus*, a member of the Apocynaceae family, produces a variety of monoterpenoid indole alkaloids (MIAs) for defense against biotic and abiotic stresses. Further investigation has revealed that certain MIAs accumulated in *C. roseus* possess potent and significant pharmacological activities, which has led to increased attention to improving their production to meet market demands [15]. Within *C. roseus*, JA has been identified as the most effective elicitor for promoting MIA production, making the JA signaling pathway a priority for potentially enhancing MIA accumulation in plants [16]. While the role of JA signaling in stimulating MIA production has been established for many years, limited research has aimed to optimize plant growth and MIA yield simultaneously. Given that JA prioritizes plant defense over growth by interfering with gibberellin signaling [17], engineering JA signaling through targeted manipulation of appropriate components in the transduction pathway is necessary for efficiently optimizing JA signaling to maximize MIA yields without compromising plant growth and productivity.

In this study, we have identified a putative JA transporter, CrJAT1 (Genbank No. OR905578), in *C. roseus* according to co-expression and phylogeny analysis for examining its impacts on the JA signaling transduction and its effect on the biosynthesis of defensive MIAs. Our results suggest that CrJAT1 can regulate the JA signaling pathway, thereby increasing the accumulation of MIAs in *C. roseus* without compromising plant growth. These findings suggest that *CrJAT1* could be a promising target for molecular breeding aimed at increasing the productivity of MIAs in *C. roseus*.

## 2. Materials and Methods

### 2.1. Plant Materials

*C. roseus* (L.) G. Don, Little delicata seeds were geminated on wet filter paper in a growth chamber under a 12-h photoperiod at 25 °C for one week. The seedlings were transplanted into pots containing soil after this initial growth period and then cultivated in a greenhouse under a 12-h light/12-h dark cycle and maintained at 25 °C.

### 2.2. Co-Expression and Phylogeny Analysis

Twelve *C. roseus* transcriptome databases (CRA_AB, CRA_AC, CRA_AD, CRA_AF, CRA_AG, CRA_AH, CRA_AI, CRA_AJ, CRA_AK, CRA_AL, CRA_AM, and CRA_AN) from the Medicinal Plant Genomics Resource (MPGR) website (http://mpgr.uga.edu/, accessed on 1 July 2021) were selected for the analysis. The databases contain transcripts from suspension cultures treated with methyl MeJA, seedlings treated with MeJA, and leaves at different developmental stages.

To identify homologs of jasmonic acid (JA) biosynthesis and transport genes in *C. roseus*, protein sequences of lipoxygenase (AtLOX), allene oxide synthase (AtAOS), OPDA reductase3 (AtOPR3), jasmonoyl isoleucine synthase (AtJAR1), and jasmonate transporter1 (AtJAT1) were retrieved from the TAIR database (https://www.arabidopsis.org/, accessed on 1 July 2021). These sequences were used as queries in a BLASTp search against the *C. roseus* genome on the MPGR website, using a 50% identity match threshold and an e-value of 1 × 10^−5^.

The homologs were then analyzed for co-expression with four selected JA-responding MIA biosynthesis genes (*CrTDC*, *CrLAMT*, *CrSTR*, and *CrNMT*). Co-expression was assessed by calculating the Pearson correlation coefficient (PCC) between expression levels of the MIA biosynthesis genes and the JA biosynthesis/transport homologs across all transcriptome databases. Genes with an absolute PCC value above 0.6 were considered co-expressed.

### 2.3. Total RNA Extraction and Molecular Cloning of CrJAT1

*C. roseus* leaves were harvested and immediately frozen in liquid nitrogen. The leaves were then ground to a fine powder using a mortar and pestle. Total RNA was extracted from the leaf powder using TRIzol reagent (Invitrogen, Shanghai, China) according to the manufacturer’s instructions. cDNAs were generated from the total RNA via reverse transcription using MMLV reverse transcriptase (Takara, Beijing, China).

The cDNAs were then used as a template to clone the *CrJAT1* gene (Genbank No. OR905578) via PCR with PrimeSTAR Max DNA polymerase (Takara, Beijing, China) by specific primers (*CrJAT1*-F and *CrJAT1*-R; see Table S1 for sequences). The resulting PCR product was gel-purified and ligated into the pGEM-T easy vector (Promega, Beijing, China) following A-tailing of the PCR product with ExTaq DNA polymerase (Takara, Beijing, China) to produce plasmid T-*CrJAT1*.

### 2.4. Overexpression and Virus-Induced Gene Silencing (VIGS) of CrJAT1 in C. roseus

The full-length coding region of *CrJAT1* was PCR-amplified using T-*CrJAT1* as a template and specific primers by adding *Xba*I/*Bam*HI restriction sites at both ends and then cloned to pGEM-T easy vector. The *CrJAT1* fragment with *Xba*I/*Bam*HI cohesive ends was obtained by *Xba*I/*Bam*HI double digestion and ligated to binary vector pBIGD (modified from pBI121 plasmid by deleting the *GUS* gene) pre-digested with *Xba*I/*Bam*HI under control of 35S promoter and NOS terminator to produce plasmid pBIGD-*CrJAT1*. For overexpression of *CrJAT1* in *C. roseus* leaves, the plasmid pBIGD-*CrJAT1* was transformed into *Agrobacterium tumefaciens* strain GV3101 for plant infection. The *Agrobacterium* transformants were grown in 50 mL of LB medium containing 50 μg/mL kanamycin, 10 mM MES, and 20 μM acetosyringone at 28 °C overnight and harvested by centrifugation. The collected cells were then resuspended in 5 mL of infiltration buffer (10 mM MgCl2, 20 mM MES, and 200 μM acetosyringone) for a 3-h incubation with shaking at 28 °C, after which they were ready for plant transformation. The stem of the 4-wk-old plant just below the apical meristem was wounded with the needle of a syringe containing *Agrobacterium* transformants for the infection, according to a method developed previously [18]. Three weeks after the infection, the newly grown leaf pairs 1 and 2 from the top of the plants, which were grown out from the transformed meristem, were harvested for gene expression and MIA accumulation analysis.

For performing the VIGS experiment, a 504-bp *CrJAT1* fragment was PCR-amplified by adding *Kpn*I/*Xho*I restriction sites at both ends with specific primers and then ligated to pGEM-T easy vector. The *CrJAT1* fragment was obtained by *Kpn*I/*Xho*I double digestion followed by gel purification and mobilized to pTRV2 vector pre-digested with *Kpn*I/*Xho*I to produce the plasmid pTRV2-*CrJAT1*. The pTRV1, pTRV2, and pTRV2-*CrJAT1* plasmids were transformed into *A*. *tumefaciens* strain GV3101, and the VIGS approach was achieved on 6-wk-old plants with three true leaf pairs, according to a previously developed method [19].

### 2.5. Gene Expression and MIA Accumulation Analysis

Total RNA extraction was conducted using a previously established method to evaluate the relative levels of gene expression in *C. roseus*. The first-strand cDNA was synthesized by MMLV reverse transcriptase using 2 μg of total RNA as the template and 2 μL of oligo dT primer (10 μM) for real-time PCR analysis. The expression of the target genes was determined by real-time PCR using the appropriate cDNA templates and primers (refer to Table S1). The expression levels of four essential genes involved in MIA biosynthesis (*CrTDC*, MG748691; *CrLAMT*, EU057974; *CrSTR*, X61932; and *CrNMT*, HM584929) were analyzed to evaluate the effects of CrJAT1 on MIA biosynthesis in *C. roseus*. The expression levels were calculated using the 2^−ΔΔCt^ method, and the expression of the *CrActin1* gene was used as a normalization control.

For MIA accumulation analysis, fresh leaves were crushed in a tissue lyser, and MIAs were extracted with methanol. The extracts were dried in a SpeedVac and then dissolved in 500 μL of methanol for HPLC analysis, according to previously developed methods [20].

### 2.6. Subcellular Localization Analysis of CrJAT1

For the vector construction, both *GFP* (with or without stop codon) and *CrJAT1* were PCR-amplified with specific primers (listed in Table S1) carrying *Xba*I/*Bam*HI and *Bam*HI/*Kpn*I at both ends of *GFP* and *CrJAT1* fragments. Then, the produced *GFP* (without stop codon) and *CrJAT1* fragments were mobilized to pBIGD vector under the control of 35S promoter and NOS terminator to produce the plasmid pBIGD-*GFP*-*CrJAT1* for transforming onion epidermal cells. For constructing the control plasmid, the *GFP* (with stop codon) fragment was PCR-amplified with specific primers (listed in Table S1) carrying *Xba*I/*Bam*HI restriction sites at both ends of *GFP* and then mobilized to pBIGD vector to produce the plasmid pBIGD-*GFP* for transforming onion epidermal cells as the control. Both pBIGD-*GFP* and pBIGD-*GFP*-*CrJAT1* were transformed into *A*. *tumefaciens* strain GV3101 to treat onion epidermal cells, according to the method developed by Sun et al. [21]. GFP fluorescence was examined with a Zeiss LSM 880 confocal microscope (Carl Zeiss GmbH, Jena, Germany) at an excitation wavelength of 488 nm, and the signal was detected at wavelengths 493–598.

## 3. Results

### 3.1. Identification of JA Signal Modulator Co-Expressed with MIA Biosynthesis Genes in C. roseus

**Figure 1:**
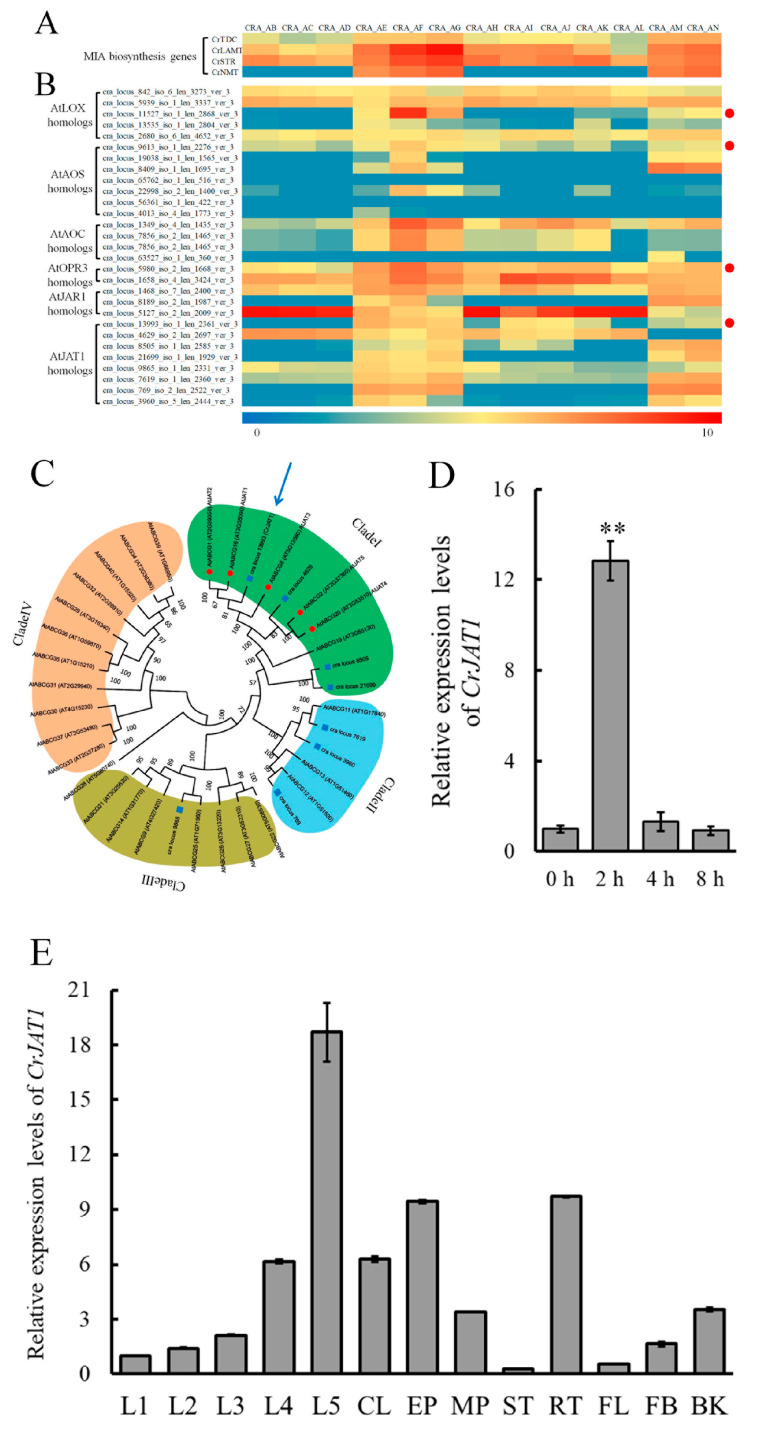
Identification of CrJAT1 in *C. roseus*. (**A**) Heatmap of expression data for essential MIA biosynthesis genes (*CrTDC*, *CrLAMT*, *CrSTR*, and *CrNMT*). (**B**) Heatmap of expression data for JA biosynthesis and transport genes. Genes co-expressed with MIA biosynthesis genes are indicated by red dots. The color scales with Z scores below the heat map indicate gene expression levels. The low and high transcript abundance are indicated by blue and red colors. (**C**) Phylogenetic analysis of JAT homologs in *C. roseus* with all ATP-binding cassette transporter G proteins (ABCG) transporters in *A. thaliana*. Blue squares indicate *C. roseus* JAT homologs and red circles indicate *A. thaliana* ABCG transporters clustered with AtJAT1 (AtABCG16). The blue arrow signifies the location of CrJAT1 according to the phylogenetic analysis. (**D**) Relative expression levels of *CrJAT1* in response to treatment with 20 μM exogenous methyl jasmonate (MeJA). (**E**) Spatial–temporal expression patterns of *CrJAT1* (L1–5: leaf pair 1–5, Leaves from top to bottom of the plants, with the uppermost leaf pair 1 being approximately 1 week old; CL: Cotyledon; EP: Leaf epidermis; MP: Leaf mesophyll cells; ST: Stems; RT: Roots; FL: Flowers; FB: Flower buds; BK: Barks). Results were normalized to *CrActin1* and are shown relative to the level in 0 h. The error bars represent standard errors from three biological replicates and each replicate contains 10 leaves from 10 independent plants of the same size and developmental age. Statistical significance was assessed with Student’s *t*-test (** *p* < 0.01).

To investigate the correlation of endogenous JA signaling with MIA biosynthesis in *C. roseus*, co-expression analysis was performed between four known JA-responsive MIA biosynthesis genes (*CrTDC*, *CrLAMT*, *CrSTR*, and *CrNMT*), which are predominately expressed in the leaf epidermal cells, and homologs of genes involved in JA biosynthesis and transport. Results showed that four JA biosynthesis/transport-related genes, encoding lipoxygenase (CrLOX), allene oxide synthase (CrAOS), OPDA reductase (CrOPR3), and jasmonate transporter1 (CrJAT1), are co-expressed with all these selected MIAs biosynthesis genes in *C. roseus* (Figure 1A,B). Given that these four genes are co-expressed with genes involved in MIA biosynthesis, it is possible that they play a significant role in modulating MIA biosynthesis in *C. roseus* by regulating the JA signaling pathway. Among the four genes, *CrJAT1* was selected for further study since it modulates JA signaling via translocating pre-existing JA molecules without stimulating new JA biosynthesis, thus possibly circumventing the generation of excessive JA signals capable of suppressing plant growth and development. To determine if the identified CrJAT1 is orthologous to AtJAT1 in *A. thaliana*, phylogenetic analysis was conducted comparing all known ABCG transporter proteins in *A. thaliana* to possible JAT1 homologs in *C. roseus*. The analysis demonstrated that CrJAT1, co-expressed with MIA biosynthesis genes, clustered closely with ABCG16 (AtJAT1) and ABCG1 (Figure 1C) in *A. thaliana*. A further experiment demonstrated that *CrJAT1* is responding to MeJA treatment (Figure 1D), indicating that the newly identified *CrJAT1* likely responds to JA signals to redistribute JAs at intra/inter-cellular levels for feedback regulating JA signaling, thereby modulating MIA biosynthesis in *C. roseus*. To characterize the expression pattern of *CrJAT1* in *C. roseus*, we conducted real-time PCR analysis on various tissues at different developmental stages (Figure 1E). The highest *CrJAT1* transcript levels were found in the fifth pair of mature leaves (L5), while the lowest expression was observed in stem tissue. Within leaves, *CrJAT1* is predominantly expressed in the epidermis and shows increasing expression as leaves mature through development. These data indicate that *CrJAT1* exhibits spatial and developmental regulation at both the tissue and cellular levels in *C. roseus*.

### 3.2. CrJAT1 Localizes at Plasma Membrane and Nuclear Envelope to Regulate JA Signaling in C. roseus

**Figure 2:**
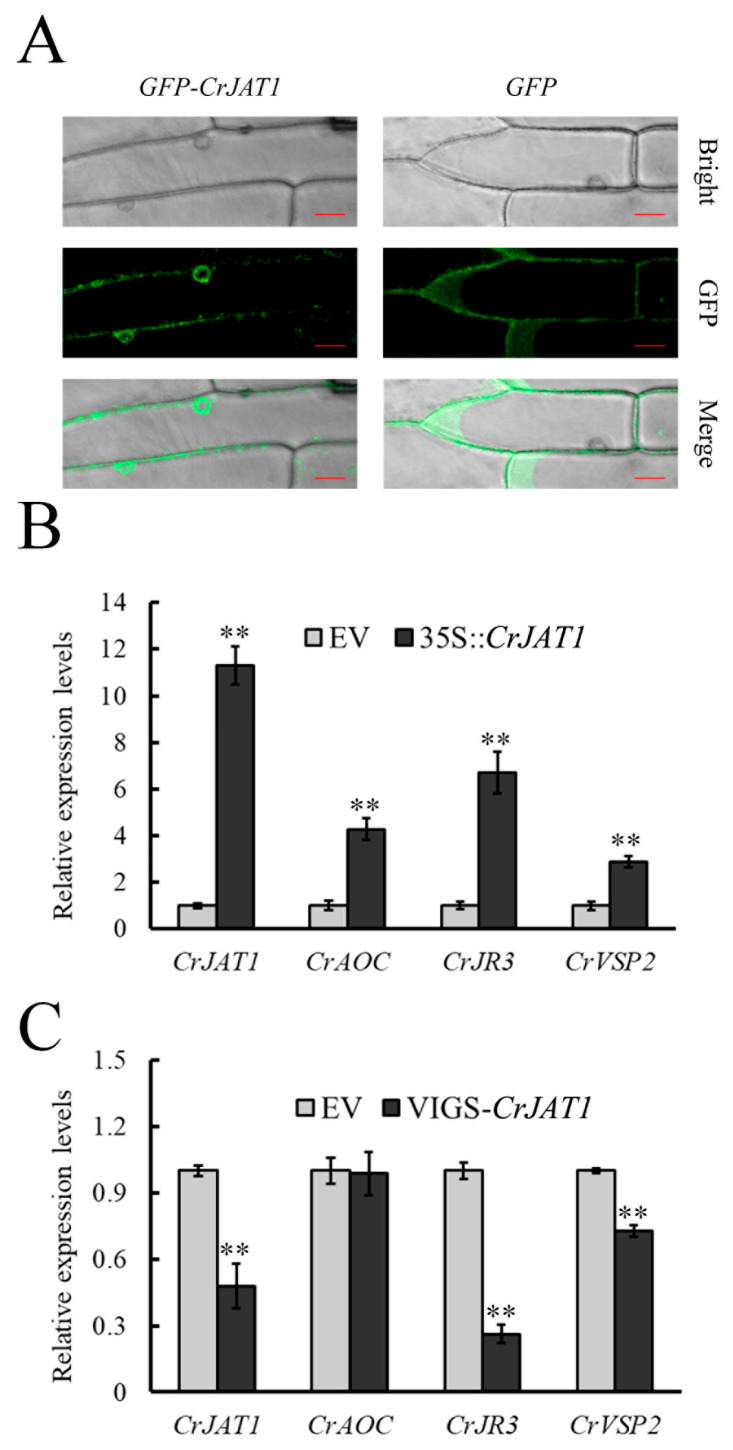
CrJAT1 is involved in regulating JA signaling. (**A**) Subcellular localization of CrJAT1 in onion epidermal cells. Scale bars, 50 μm. (**B**) Relative expression levels of *CrJAT1* and JA-responding genes in *CrJAT1*-overexpressed leaves. (**C**) Relative expression levels of *CrJAT1* and JA-responding genes in *CrJAT1*-silenced leaves. Results were normalized to *CrActin1* and are shown relative to the level in empty vector control leaves. The error bars represent standard errors from three biological replicates and each replicate contains 10 leaves from 10 independent plants of the same size and developmental age. Statistical significance was assessed with Student’s *t*-test (** *p* < 0.01).

Since the synthesized JA-Ile should be transported to the nucleus for activating JA signaling, we examined the subcellular localization of CrJAT1 in onion epidermal cells. Results showed that CrJAT1 localized both plasma membrane and nuclear envelope (Figure 2A), which is consistent with the localization sites of AtJAT1 in *A. thaliana*.

To characterize the function of the identified CrJAT1 in JA signaling, we overexpressed *CrJAT1* in *C. roseus* leaves to assess its effects on the expression of three known JA-responsive genes (*CrAOC*, *CrJR3*, and *CrVSP2*). The results showed that the expression of the three JA-responsive genes was significantly induced in *CrJAT1*-overexpressed leaves compared to the empty vector control, indicating that *CrJAT1* overexpression activates JA signaling (Figure 2B). To further validate the role of CrJAT1 in regulating JA signaling, we silenced *CrJAT1* expression in *C. roseus* leaves and measured the expression of the same three JA-responsive genes. The results demonstrated that, with the exception of *CrAOC*, expression of the other two genes (*CrJR3* and *CrVSP2*) was significantly down-regulated in *CrJAT1*-silenced leaves compared to the empty vector control (Figure 2C). Our findings suggest that CrJAT1 is essential in modulating JA signaling, independent of direct effects on de novo JA biosynthesis in *C. roseus*.

### 3.3. Effect of Overexpressing CrJAT1 on MIA Biosynthesis in C. roseus

**Figure 3:**
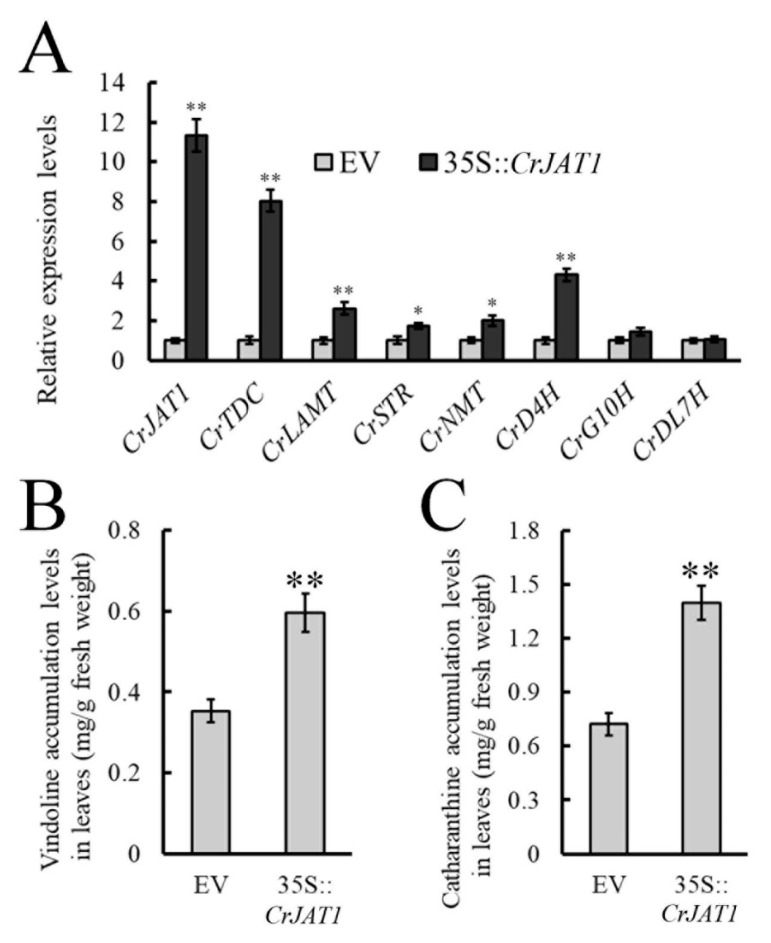
The effects of *CrJAT1* overexpression on the expression of the MIA biosynthetic genes (**A**) and the accumulation of vindoline (**B**) and catharanthine (**C**) in *C. roseus* leaves. Real-time PCR results were normalized to *CrActin1* and are shown relative to the level in empty vector control leaves. The error bars represent standard errors from three biological replicates and each replicate contains 10 leaves from 10 independent plants of the same size and developmental age. Statistical significance was assessed with Student’s *t*-test (* *p* < 0.05, ** *p* < 0.01).

In order to further study the effect of the CrJAT1-mediated endogenous JA signaling pathway on MIA biosynthesis, we examined both pathway gene expression and MIA accumulation in *CrJAT1*-overexpressed leaves. Seven known JA-responsive MIA biosynthesis genes were selected based on previous gene expression studies, and their transcriptional responses were investigated following *CrJAT1* overexpression. As visualized in Figure 3A, the expression levels of five selected MIA biosynthesis pathway genes, *CrTDC*, *CrLAMT*, *CrSTR*, *CrNMT*, and *CrD4H*, were remarkably upregulated as a direct result of elevated *CrJAT1* expression. This dramatic induction of significant MIA biosynthetic genes demonstrated that *CrJAT1* overexpression strongly activates the JA signaling cascade to stimulate MIA production. To further confirm the functional impact of CrJAT1 on actual MIA accumulation, we directly measured the endogenous levels of two essential MIAs, vindoline and catharanthine, in *CrJAT1*-overexpressed leaves. The results showed that vindoline levels were 1.68-fold higher (Figure 3B) while catharanthine levels increased 1.93-fold more (Figure 3C) than control leaves without overexpressing *CrJAT1*. These findings provide compelling evidence that the CrJAT1 protein plays a pivotal role in positively regulating endogenous JA signaling networks to subsequently modulate the biosynthesis of valuable MIAs in *C. roseus* at both the transcriptional and metabolic levels.

### 3.4. Effect of Silencing CrJAT1 on MIA Biosynthesis in C. roseus

**Figure 4:**
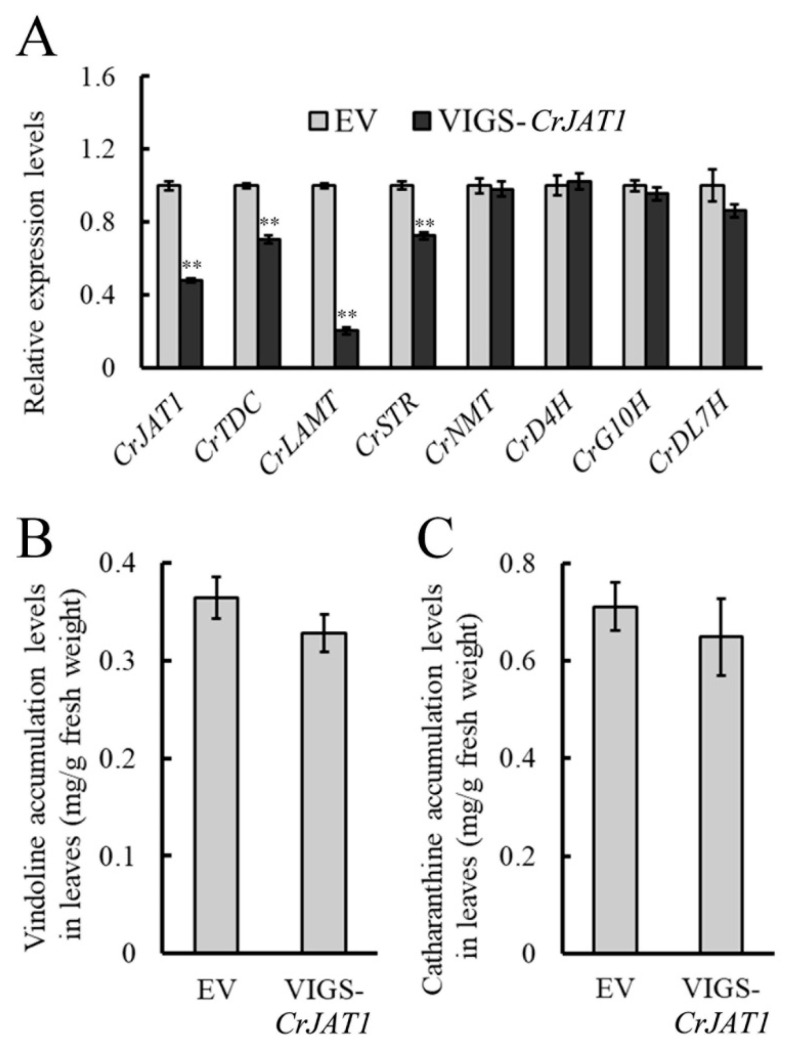
The effects of *CrJAT1* silencing on the expression of the MIA biosynthetic genes (**A**) and the accumulation of vindoline (**B**) and catharanthine (**C**) in *C. roseus* leaves. Real-time PCR results were normalized to *CrActin1* and are shown relative to the level in empty vector control leaves. The error bars represent standard errors from three biological replicates and each replicate contains 10 leaves from 10 independent plants of the same size and developmental age. Statistical significance was assessed with Student’s *t*-test (** *p* < 0.01).

To verify the role of CrJAT1 in regulating MIA biosynthesis in *C. roseus*, a VIGS approach was utilized to silence the expression of *CrJAT1*. The expression of three MIA biosynthetic genes, *CrTDC*, *CrLAMT*, and *CrSTR*, was found to be downregulated in *CrJAT1*-silenced leaves, indicating that the expression level of *CrJAT1* truly affects MIA biosynthesis in *C. roseus* (Figure 4A). However, no significant differences were observed in the accumulation of vindoline and catharanthine between treatments (Figure 4B,C), which may be due to low expression of pathway genes and pathway compartmentalization. Given the inherently low baseline accumulation levels of these MIAs in plants, it presents a challenge to observe further decreases in MIAs that are already present at such low concentrations. Therefore, although silencing of *CrJAT1* leads to repressed endogenous JA signaling, the compartmentalized pattern of MIA biosynthesis and low basal MIA accumulation levels do not result in substantial changes in vindoline or catharanthine production between *CrJAT1*-silenced leaves and empty vector control leaves in *C. roseus*.

## 4. Discussion

In addition to enhancing plant resistance to biotic stresses, some MIAs accumulated in *C. roseus* have pharmaceutical applications, such as the anticancer alkaloids vinblastine and vincristine. However, the MIA biosynthesis genes exhibit strictly cell-specific expression patterns with the involvement of at least three cell types (the internal phloem associated parenchyma (IPAP), laticifers/idioblasts, and epidermal cells) and six intracellular compartments (plastid, chloroplast, vacuole, nucleus, endoplasmic reticulum, and cytosol) [22,23]. This complex spatiotemporal regulation likely contributes to the low accumulation of these valuable compounds in *C. roseus*, limiting their utilization for clinical therapy. Further elucidation of the regulatory mechanisms governing MIA biosynthesis is thus imperative to facilitate the efficient production of these pharmaceuticals for improved therapeutic applications.

Jasmonates have been shown to effectively regulate the biosynthesis of MIAs in *C. roseus*. As a result, modulating endogenous JA signaling through genetic or chemical means has attracted interest in boosting MIA production, as the large-scale application of exogenous JAs is not feasible for actual industrial production. To date, numerous transcription factors that mediate JA signaling to regulate MIA biosynthesis have been identified and proposed for increasing MIA accumulation via genetic engineering [24,25,26]. However, these transcription factors target only partial MIA biosynthetic genes, limiting their potential application, since MIA biosynthesis is strictly controlled spatially and temporally [22,23]. Given this complex biosynthetic process, simplistic expression modulation of selected transcription factors may be insufficient to effectively enhance MIA yields in *C. roseus*. A deeper understanding of transcriptional and metabolic networks underlying MIA production is warranted to develop strategies to optimize their yields for industrial production and therapeutic applications.

JA signaling regulates both the expression of MIA biosynthetic genes and intermediate transporter genes, effectively overcoming limitations imposed by pathway compartmentation and promoting overall MIA accumulation [19,27]. Therefore, modulating the entire JA signaling transduction pathway could enable a high accumulation of MIAs in *C. roseus*. However, JAs play a role in coordinating the trade-off between growth and defense, as mutants with decreased JA signaling exhibit increased stature/growth but decreased pathogen resistance [14]. Excessive JA signaling severely inhibits plant growth [28,29], resulting in low MIA productivity. This demonstrates that solely upregulating endogenous JA levels is insufficient for improving MIA yields in *C. roseus*. A balanced approach is needed to optimize signaling without compromising plant fitness. Rather than focusing solely on modulating endogenous JAs accumulation levels, dynamic regulation of the JA signaling network in a coordinated way may achieve higher, more sustainable production.

Recent work on *A. thaliana* indicates that an ABC transporter, AtJAT1, could balance plant growth and defense by regulating the levels of JA and its biologically active form, JA-Ile, within plant cells. AtJAT1 transports JA-Ile from the cytosol into the nucleus to activate the expression of JA-responsive genes without de novo JA biosynthesis [14]. This transport of JA-Ile is essential for regulating the tradeoff between plant growth and defense by activating or deactivating JA signaling. AtJAT1 also exports JA extracellularly to deplete the cytosolic JA pool, deactivating signaling and triggering systemic immunity. Interestingly, AtJAT1 preferentially imports JA-Ile into the nucleus when cytosolic JA/JA-Ile levels are low, but exports JA outside cells when cytosolic JA/JA-Ile levels are high. This dual transport activity ensures proper tuning of JA signaling to protect the plant from stresses without inhibiting growth. Precise subcellular redistribution of signaling molecules, rather than their de novo biosynthesis, may effectively balance specialized metabolism and growth.

In this study, we aimed to identify the most suitable modulator of JA signaling as a target for potential molecular breeding to increase MIA productivity. Through co-expression and phylogeny analyses, we identified a *C. roseus* homolog of AtJAT1, designated CrJAT1. Overexpression of *CrJAT1* clearly activated JA signaling and significantly enhanced MIA accumulation levels without inhibiting plant growth. In contrast, exogenous JA spraying led to a marked reduction in fresh weight of *C. roseus* leaves (Figure S1). In *A. thaliana*, JAT1 has been shown to play a role in autonomously regulating the balance between growth and defense by activating and deactivating JA signaling. Given the results of our current study, CrJAT1 may serve as an effective means of modulating JA signaling to enhance MIA production in *C. roseus*. By tuning JA pathway activity through CrJAT1, we may be able to optimize metabolic flux towards the biosynthesis of economically important MIAs in this species. However, further experimental validation is still needed to precisely characterize the JA transport activity of CrJAT1. Overall, this study provides a promising candidate for modulation of specialized metabolism through targeted signaling adjustment rather than growth compromise in *C. roseus*.

## Data Availability

Data will be provided on reasonable request to the corresponding author. The data are not publicly available due to the data also forms part of an ongoing study.

## Supplementary Materials

The following supporting information can be downloaded at: https://www.mdpi.com/article/10.3390/genes15030324/s1, Figure S1: Fresh weight of empty vector control leaves (EV), *CrJAT1*-overexpressed leaves (35S::*CrJAT1*), and empty vector control leaves treated with 20 µM MeJA (EV+MeJA); Table S1: List of primers used in this work.
