# Supplementary material for: CrJAT1 Regulates Endogenous JA Signaling for Modulating Monoterpenoid Indole Alkaloid Biosynthesis in Catharanthus roseus
Source: Genes (Basel). 2024 Mar 2;15(3):324. doi: 10.3390/genes15030324 (PMC10970522; doi:10.3390/genes15030324)
Supplement: Supplementary file 1 [file genes-15-00324-s001.zip › genes-2875419-SI.pdf]

Supplementary Information

CrJAT1 regulates endogenous JA signaling for modulating monoterpenoid indole alkaloid biosynthesis in *Catharanthus roseus*

Mengxia Zhang[1], Bingrun Yang[1], Yanyan Wang[1], Fang Yu[1, 2*]

[1] School of Biological Engineering, Dalian Polytechnic University, Dalian, 116034, China

[2] College of Bioscience and Biotechnology, Shenyang Agricultural University, Shenyang, 110866, China

*Corresponding author: Fang Yu

E-mail address: yufang@dlpu.edu.cn or fyu0506@gmail.com

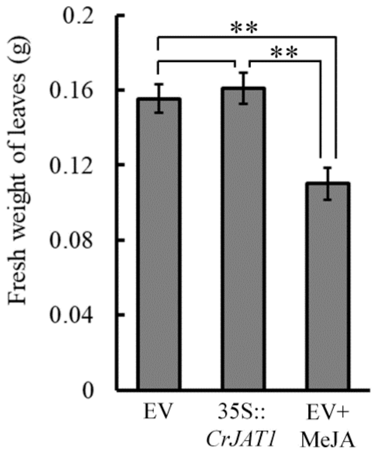

Figure S1. Fresh weight of empty vector control leaves (EV), *CrJAT1*-overexpressed leaves (35S::*CrJAT1*), and empty vector control leaves treated with 20 µM MeJA (EV+MeJA). The plant stem just below the apical meristem were treated with *Agrobacterium* GV3101 containing either empty vector or *CrJAT1*-overexpression vector. One week after *Agrobacterium* treatments, half of EV plants were sprayed with 20 µM MeJA every three days. Two weeks after MeJA treatment, two leaf pairs from the top of plants were harvested to measure fresh weight. The error bars represent standard errors from three biological replicates and each replicate contains 10 leaves from 10 independent plants with the same size and developmental age. Statistical significance was assessed with Student's t-test (**p<0.01).
